# Vaccination Decreases the Infectious Viral Load of Delta Variant SARS-CoV-2 in Asymptomatic Patients

**DOI:** 10.3390/v14092071

**Published:** 2022-09-18

**Authors:** Jessica A. Plante, Rafael R. G. Machado, Brooke M. Mitchell, Divya P. Shinde, Jordyn Walker, Dionna Scharton, Allan McConnell, Nehad Saada, Jianying Liu, Bilal Khan, Rafael K. Campos, Bryan A. Johnson, Vineet D. Menachery, Corri B. Levine, Ping Ren, Susan L. F. McLellan, Kenneth S. Plante, Scott C. Weaver

**Affiliations:** 1 World Reference Center for Emerging Viruses and Arboviruses, University of Texas Medical Branch, Galveston, TX 77555, USA; 2 Department of Microbiology and Immunology, University of Texas Medical Branch, Galveston, TX 77555, USA; 3 Department of Microbiology, Institute of Biomedical Sciences, University of Sao Paulo, Sao Paulo 05508-900, CEP, Brazil; 4 Institute of Infectious Diseases, Shenzhen Bay Laboratory, Shenzhen 518071, China; 5 Department of Molecular Pathology, Dow University of Health Sciences, Karachi 74200, Pakistan; 6 Institute for Human Infections and Immunity, University of Texas Medical Branch, Galveston, TX 77555, USA; 7 Department of Internal Medicine, Division of Infectious Diseases, University of Texas Medical Branch, Galveston, TX 77555, USA; 8 Department of Pathology, University of Texas Medical Branch, Galveston, TX 77555, USA

**Keywords:** SARS-CoV-2, COVID-19, delta, B.1.617.2, vaccination, breakthrough, IgA

## Abstract

The Delta variant of SARS-CoV-2 has caused many breakthrough infections in fully vaccinated individuals. While vaccine status did not generally impact the number of viral RNA genome copies in nasopharyngeal swabs of breakthrough patients, as measured by Ct values, it has been previously found to decrease the infectious viral load in symptomatic patients. We quantified the viral RNA, infectious virus, and anti-spike IgA in nasopharyngeal swabs collected from individuals asymptomatically infected with the Delta variant of SARS-CoV-2. Vaccination decreased the infectious viral load, but not the amount of viral RNA. Furthermore, vaccinees with asymptomatic infections had significantly higher levels of anti-spike IgA in their nasal secretions compared to unvaccinated individuals with asymptomatic infections. Thus, vaccination may decrease the transmission risk of Delta, and perhaps other variants, despite not affecting the amount of viral RNA measured in nasopharyngeal swabs.

## 1. Introduction

Vaccination is a critical tool for reducing the disease burden of COVID-19, the disease caused by SARS-CoV-2. The BNT162b2 (Pfizer-BioNTech) and mRNA-1273 (Moderna) mRNA vaccines, as well as the Ad26.COV2.S (Johnson & Johnson–Janssen) adenovirus-vectored vaccine, express the spike protein of the original strain of SARS-CoV-2 to elicit an immune response. When the original and alpha variant of SARS-CoV-2 were predominant, the vaccines effectively prevented infection, as well as the development of severe COVID-19 symptoms [1,2]. The Delta variant, however, was associated with many breakthrough infections in fully vaccinated individuals [3]. Although the vaccines were still largely protective against severe disease and death from Delta [2], this finding raised the question of whether vaccination is still a useful method for reducing SARS-CoV-2 transmission.

Cycle threshold (Ct) values from PCR-based tests for SARS-CoV-2 reflect the quantity viral RNA, most frequently in nasopharyngeal (NP) swabs. For patients infected with the Delta variant of SARS-CoV-2, the impact of vaccination on Ct values was variable, with any protective effect decreasing rapidly after inoculation [3,4]. However, this Ct analysis does not necessarily reflect the infectious viral load that results in transmission. In this study, we compare the Ct values, infectious viral load, and anti-spike IgA levels in NP swabs from both unvaccinated and fully vaccinated patients infected with the Delta variant of SARS-CoV-2. We conclude that vaccination decreases the amount of infectious virus following SARS-CoV-2 Delta infection in asymptomatic individuals, potentially due to increased levels of secreted anti-spike IgA antibodies.

## 2. Materials and Methods

### 2.1. Human Nasopharyngeal Swab and Patient Data Collection

NP swabs were collected in universal or viral transport media for routine diagnostic purposes at the Galveston Campus Clinical Laboratory of the University of Texas Medical Branch (Galveston, TX, USA) between 2 June and 11 August 2021. All assays were performed on the liquid universal or viral transport media in which the NP swab was submerged and agitated. Samples were collected from asymptomatic individuals being screened due to suspected exposure, hospital admission for a non-COVID-19-related reason, or work or travel requirements. Leftover material from samples that tested positive for SARS-CoV-2 by RT-PCR using the Panther Fusion SARS-CoV-2 assay (Hologic, San Diego, CA, USA) were stored at 4 °C for 1–7 days (mean of 4) before being aliquoted and stored at −80 °C. Patient data including sample collection date, sex, race, ethnicity, vaccination status, presence or absence of signs and symptoms both at the time of sample collection and for one month following sample collection, and the presence of pre-existing co-morbidities (cancer, cerebrovascular disease, chronic kidney disease, COPD, corticosteroid use, diabetes, Down’s syndrome, heart conditions, HIV, neurologic conditions, other lung disease, overweight or obesity, pregnancy, sickle cell disease, smoking, substance abuse, and organ transplant) were collected through patient record reviews. Ct values were obtained from the Panther Fusion SARS-CoV-2 assay. Samples collected from incarcerated patients or patients under the age of 18 or from patients with unknown or incomplete vaccination status were excluded. Vaccination was defined as one dose of the Johnson & Johnson–Janssen vaccine or two doses of the Moderna or Pfizer–BioNTech vaccine. All work was conducted in accordance with protocol 21-0226, as authorized by the Institutional Review Board at UTMB.

### 2.2. Sequencing

RNA was extracted from NP swabs using TRIzol LS (Ambion, Carlsbad, CA, USA) according to the manufacturer’s instructions. An approximately 2345 nucleotide region of the SARS-CoV-2 spike gene was amplified using the SuperScript IV One-Step RT PCR System (Invitrogen, Carlsbad, CA, USA) with forward primer 5′-TGTTATTTCTAGTGATGTTCTTG-3′ and reverse primer 5′-GTTAAAGCACGGTTTAATTGTG-3′ [5]. Reactions were incubated for 10 min at 50 °C, 2 min at 98 °C; cycled 40 times through a three-step program of 10 s at 98 °C, 10 s at 58 °C, and 2 min at 72 °C; then incubated for 5 min at 72 °C and held at 4 °C. The resulting amplicons were purified with the QIAquick PCR Purification Kit (Qiagen, Hilden, Germany) prior to Sanger dideoxy sequencing with ABI BigDye Terminator v3.1 Ready Reaction Mix (Applied Biosystems, Austin, TX, USA). Sequencing reactions were purified with Performa DTR gel filtration cartridges (EdgeBio, Gaithersburg, MD, USA) and read on a 3500 Genetic Analyzer (Applied Biosystems, Austin, TX, USA).

### 2.3. Infectious Virus Quantification

The nasopharyngeal samples were serially diluted in DMEM (Gibco, Grand Island, NY, USA) with 1% antibiotic–antimycotic (Gibco, Grand Island, NY, USA) and allowed to infect a confluent monolayer of Vero E6 cells in a 96-well plate for 45 min at 37 °C with 5% CO_2_. Following infection, cells were overlaid with a solution of 85% MEM (Gibco, Grand Island, NY, USA) and 15% DMEM supplemented with 1% antibiotic–antimycotic and 0.85% methyl cellulose (Sigma, St. Louis, MO, USA). After approximately 30 h, the monolayers were fixed with formalin (Fisher, Pittsburgh, PA, USA) for at least 24 h. Monolayers were washed with DPBS (Sigma, St. Louis, MO, USA) and incubated in permeabilization buffer consisting of DPBS supplemented with 0.1% BSA (Sigma, St. Louis, MO, USA) and 0.1% saponin (Sigma, St. Louis, MO, USA) for 30 min at room temperature. Permeabilization buffer was removed, and monolayers were incubated overnight at 4 °C with rabbit polyclonal antibody against SARS-CoV N protein (Dr. Shinji Makino, Department of Microbiology & Immunology, UTMB, Galveston, TX, USA) diluted in permeabilization buffer. Excess antibody was removed by washing with DPBS, and monolayers were incubated for one hour at room temperature with HRP-conjugated goat anti-rabbit IgG (Cell Signaling, Beverly, MA, USA) diluted in permeabilization buffer. Excess antibody was washed away with DPBS, and foci were stained using KPL TrueBlue Peroxidase Substrate (SeraCare, Milford, MA, USA). Once foci were visible under a light microscope, excess substrate was removed and the monolayers were washed with water. Wells were imaged using the Cytation7 Imagining Reader (BioTek, Winooski, VT, USA). Foci were counted manually.

### 2.4. Antibody Quantification

Residual NP suspensions were heat inactivated for 30 min at 65 °C following viral quantification and stored at −80 °C until the day of the IgA assay. Heat inactivation had a statistically significant but small impact on measured concentration, reducing the S1 standards by 1.6 pg/mL and the receptor-binding domain (RBD) standards by 1.4 pg/mL (Figure S1). Human IgA capable of binding either the S1 subunit of the SARS-CoV-2 spike protein or the RBD of the SARS-CoV-2 spike protein was quantified in undiluted NP swab material using the LEGENDplex SARS-CoV-2 Serological IgA Panel (BioLegend, San Diego, CA, USA) according to the manufacturer’s protocol. Samples were run on a LSRFortessa (BD Biosciences, Franklin Lakes, NJ, USA) by the Flow Cytometry and Cell Sorting Core Lab at UTMB (Galveston, TX, USA). Standard curve generation and sample concentration determination was carried out using the LEGENDplex Data Analysis Software Suite (BioLegend, San Diego, CA, USA).

### 2.5. Statistical Analysis

To compare the demographics of the vaccinated and unvaccinated cohorts, continuous variables (sample storage time, age, BMI) were compared by Mann–Whitney, while categorical and discrete variables (sex, race, ethnicity, number of pre-existing co-morbidities, and development of symptoms post-sample collection) were compared by Fisher’s exact test. Infectious titers and IgA concentrations were log_(10)_ converted prior to analysis. Infectious titers below the limit of detection were counted as one-half of that value for statistical and graphing purposes. Ct values, infectious titers, and IgA levels of vaccinated and unvaccinated asymptomatic patients were analyzed using Mann–Whitney tests. The impact of heat inactivation on IgA standard curve generation was assessed by comparing the linear regression of the theoretical and measured concentrations using ANCOVA. The impact of age on viral load and antibody phenotypes was evaluated by simple linear regression with Spearman’s correlation to detect significance. In all analyses, α = 0.05 was the threshold of significance. All graphing and analyses were performed in Prism for Windows v9.3.1 (GraphPad, San Diego, CA, USA), except for the Fisher’s exact tests of race and co-morbidity data, which was performed using the Simple Interactive Statistical Analysis website [6]. All raw data are available in Table S1.

## 3. Results

### 3.1. SARS-CoV-2 Quantification

Eighty-seven NP swabs were collected from patients infected with the Delta variant of SARS-CoV-2 (Table 1) as confirmed by sequencing of the spike gene. Of this population, 28 were vaccinated and 59 were unvaccinated. The vaccinated cohort was significantly older than the unvaccinated cohort, in keeping with the trend of older individuals being vaccinated at higher rates [7]. In the unvaccinated cohort, age was significantly correlated with increased viral RNA loads and decreased anti-SARS-CoV-2 IgA (Figure S2). However, there was no significant correlation between age and viral loads or IgA levels in the vaccinated cohort. In all other health (BMI, number of co-morbidities) and demographic (sex, race, and ethnicity) characteristics, as well as the number of days for which the NP swab was held at 4 °C prior to freezing at −80 °C, the vaccinated and unvaccinated populations were statistically indistinguishable. The rates of self-reported symptom development subsequent to the collection of the positive NP swab were similar in the vaccinated and unvaccinated groups. However, these data should be interpreted with caution, as they captured only self-reported symptoms from individuals seeking medical attention or advice within the University of Texas Medical Branch system; if an individual sought treatment from another healthcare system or treated their symptoms at home without the aid of a medical professional, that information would not have been captured in this dataset. No hospital admissions or fatalities were reported in the study population.

Demographic and health characteristics of the unvaccinated and vaccinated study subjects. Number of co-morbidities was calculated only for subjects with full medical records available for review (36 unvaccinated, 24 vaccinated). Continuous variables (length of sample storage at 4 °C, age, and BMI) are shown as the median value with the interquartile range and were evaluated using the Mann–Whitney test. Discrete variables (sex, race, ethnicity, number of comorbidities, and symptom development) are shown as the total number of subjects followed by the percent and were evaluated using Fisher’s exact test.

**Figure 1:**
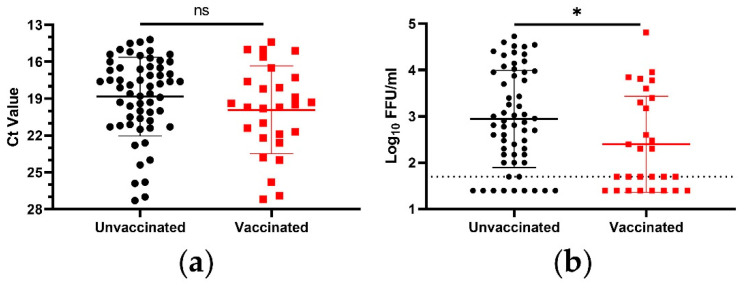
Viral load in NP swabs from asymptomatic Delta variant SARS-CoV-2 infections. (**a**) SARS-CoV-2 RNA was measured using real time RT-PCR. (**b**) Infectious SARS-CoV-2 was measured via a microtiter assay. Symbols represent individual subjects, midlines represent the mean, error bars represent the standard deviation, and dashed lines represent the lower limit of the assay. Unvaccinated and vaccinated groups were compared via Mann–Whitney test. ns = *p* > 0.05, * = *p* < 0.05.

Assay selection had a demonstrable impact on the perceived relationship between vaccination status and viral load in the NP swabs of patients asymptomatically infected with the Delta variant of SARS-CoV-2. When considering only viral RNA, vaccinated patients did trend toward lower viral loads (decrease in Ct value of 1.1 by mean, 1.6 by median) (Figure 1a). However, this trend was not statistically significant. When considering infectious virus, on the other hand, vaccination did decrease the viral load in a statistically significant manner (Figure 1b). The vaccine-dependent decrease in infectious virus was 0.5log_10_ FFU/mL, as measured by mean, and 0.9log_10_ FFU/mL, as measured by median, translating to 3.5-fold and 7.5-fold decreases, respectively. Additionally, a significantly higher proportion of the NP swabs from vaccinated individuals were at or below the detection limit of the assay: 50.0% of the NP swabs from vaccinated individuals, compared to only 18.6% from unvaccinated individuals (*p* = 0.0047, Fisher’s exact test).

### 3.2. IgA Quantification

**Figure 2:**
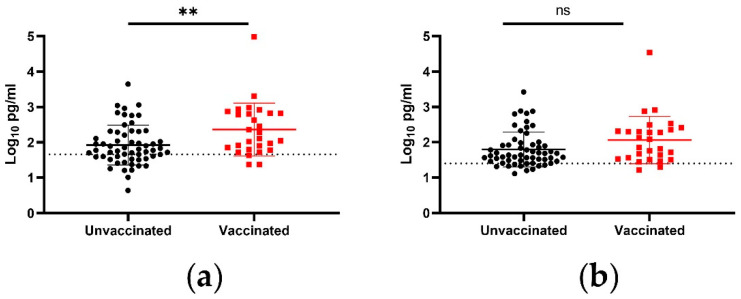
Anti-SARS-CoV-2 IgA in NP swabs from asymptomatic Delta variant SARS-CoV-2 infections. IgA capable of binding (**a**) the SARS-CoV-2 S1 protein or (**b**) the SARS-CoV-2 RBD was measured via flow cytometry. Symbols represent individual subjects, midlines represent the mean, error bars represent the standard deviation, and dashed lines represent the lower limit of the assay. Unvaccinated and vaccinated groups were compared by Mann–Whitney test. ns = *p* > 0.05, ** = *p* < 0.01.

In addition to viral load, IgA capable of binding either the S1 or the RBD of SARS-CoV-2 in nasal secretions was measured. The vaccinated subjects had a statistically significant increase in anti-S1 IgA (Figure 2a). The increase was 148 pg/mL, as measured by mean, and 95 pg/mL, as measured by median, translating to 2.8-fold and 2.5-fold increases, respectively. Vaccination was also associated with a significantly higher proportion of the NP swabs having detectable levels of anti-S1 IgA: 92.9% of vaccinated individuals, compared to 64.4% of unvaccinated individuals (*p* = 0.0044, Fisher’s exact test). While the anti-RBD IgA trended toward higher levels in vaccinated individuals, that trend approached but did not meet the threshold of statistical significance (*p* = 0.0558, Mann–Whitney test) (Figure 2b).

## 4. Discussion

The broad availability of vaccines has changed the dynamics of the COVID-19 pandemic. However, the prevalence of breakthrough infections in vaccinated individuals leaves questions about their efficacy against infection and transmission, especially against novel variants. Vaccination has been shown to decrease the recovery of infectious virus even in instances of comparable Ct values [8] and to significantly decrease infectious titers in NP swabs at days 0–5 post-symptom onset following infection with Delta variant SARS-CoV-2 [9]; however, the effect of vaccination in asymptomatic individuals was not addressed.

We demonstrated that vaccination significantly decreased the infectious titers’ NP swabs collected from asymptomatic breakthrough individuals. This result contrasts viral RNA-based results which found a modest, non-significant reduction, emphasizing the need to perform infectious assays when addressing questions of viral load. Although Ct values can be obtained without the need for biocontainment facilities and are readily available from routine diagnostic testing, infectious virus drives transmission, and its direct quantification is the most appropriate laboratory measurement to address transmission-related hypotheses. The 3.5- to 7.5-fold differences in infectious titer we saw in asymptomatic individuals may underestimate their importance to SARS-CoV-2 transmission. Approximately half of SARS-CoV-2 transmission events are predicted to originate from asymptomatic individuals [10,11]. While a symptomatic individual may reasonably be expected to isolate or take other protective measures, an asymptomatic or pre-symptomatic individual is less likely to take enhanced precautions to reduce the risk of transmission. It would be interesting to model whether (or to what degree) our reported non-sterilizing decrease in infectious viral titer impacted transmission from asymptomatic individuals during the Delta wave.

The increased levels of secreted IgA in vaccinated as opposed to unvaccinated asymptomatic individuals offers a possible explanation for the observed decrease in infectious titer without a corresponding increase in Ct value. Antibodies at the site of initial infection are important to achieving sterilizing immunity. In the case of respiratory viruses, this means that IgA in nasal secretions, which may neutralize some infectious viruses, is a key measure of immunity. The importance of IgA as a correlate of protection has been demonstrated for the influenza virus [12,13]; its routine incorporation in the evaluation of SARS-CoV-2 vaccines will be important for vaccine comparison and optimization as the pandemic continues

Because age differed significantly between our vaccinated and unvaccinated cohorts, its potential impact on the observed differences in infectious titers and IgA levels must be considered. In vaccinated individuals, age had no impact on viral load or IgA. In unvaccinated individuals, age was positively correlated with viral RNA loads and negatively correlated with anti-S1 and anti-RBD IgA, as expected [14,15,16,17]. If the lower mean age of the unvaccinated group was responsible for these observed differences, one would expect the unvaccinated group to have lower viral loads and higher IgA levels. However, we observed the opposite: unvaccinated individuals had higher viral loads and lower levels of IgA. We therefore believe that vaccination is far more likely to be the source of our observed differences in infectious viral loads.

While there have been other examinations of infectious virus following SARS-CoV-2 infection [9,18,19], our study’s combination of vaccinated and unvaccinated asymptomatic patients offers a unique window into whether and how vaccination may have impacted transmission of the SARS-CoV-2 Delta variant within this population. Limitations include sample size, lack of data on whether subjects were previously infected, and the restriction of samples to NP swabs. Additional data would allow for the consideration of other potentially important factors, such as the length of time between vaccination and infection, and additional samples and power might render the trend of higher Ct values in vaccinated individuals statistically significant. Additional patient data, such as whether an individual had previously been infected with SARS-CoV-2, might yield additional insights into the mechanism(s) of the observed differences. It is also possible that a similar study conducted on lung tissue or bronchoalveolar lavages might yield a different result. Finally, although our focus forming assays in Vero cells do measure infectious virus, infectivity in a Vero cell and infectivity in a human airway are not necessarily identical. Measures of infectivity in other models that may be more reflective of the human airway, such as immortalized or primary human airway epithelial cells cultured with an air–liquid interface, might be illuminating.

## Figures and Tables

**Table 1:** Characteristics of the Study Population.

	Unvaccinated	Vaccinated	*p*-Value
**Total Number of Subjects**	59	28	N/A
**Days of Sample Storage at 4 °C Prior to Freezing at −80 °C**	5 (3–5)	5 (3–6)	0.7615
**Years of Age**	40.1 (30.1–49.0)	48.2 (35.3–69.8)	0.0063
**BMI**	31.8 (28.5–37.4)	33.6 (30.0–39.6)	0.2950
**Sex**			
Female	30 (50.9)	14 (50.0)	>0.9999
Male	29 (49.2)	14 (50.0)	
**Race**			
Black or African American	7 (11.9)	4 (14.3)	0.3481
Caucasian/White	52 (88.1)	23 (82.1)	
Asian	0 (0.0)	1 (3.6)	
**Ethnicity**			
Hispanic or Latino	15 (25.4)	6 (21.4)	0.7922
Not Hispanic or Latino	44 (74.6)	22 (78.6)	
**Number of Comorbidities**			
0	3 (8.3)	2 (8.3)	0.3477
1	18 (50.0)	6 (25.0)	
2	7 (19.4)	8 (33.3)	
3	5 (13.9)	6 (25.0)	
4–5	3 (8.3)	2 (8.3)	
**Reported Symptom Development After Sample Collection**	14 (23.7)	7 (25.0)	>0.9999

## Data Availability

All raw data are available in Table S1.

## Supplementary Materials

The following supporting information can be downloaded at: https://www.mdpi.com/article/10.3390/v14092071/s1, Figure S1: Impact of Heat Inactivation on Measured IgA; Figure S2: Impact of Age on Viral Load and IgA; Table S1: Raw Data.
