# Supplementary figures and images for: Vaccination Decreases the Infectious Viral Load of Delta Variant SARS-CoV-2 in Asymptomatic Patients
Source: Viruses. 2022 Sep 18;14(9):2071. doi: 10.3390/v14092071 (PMC9503182; doi:10.3390/v14092071)

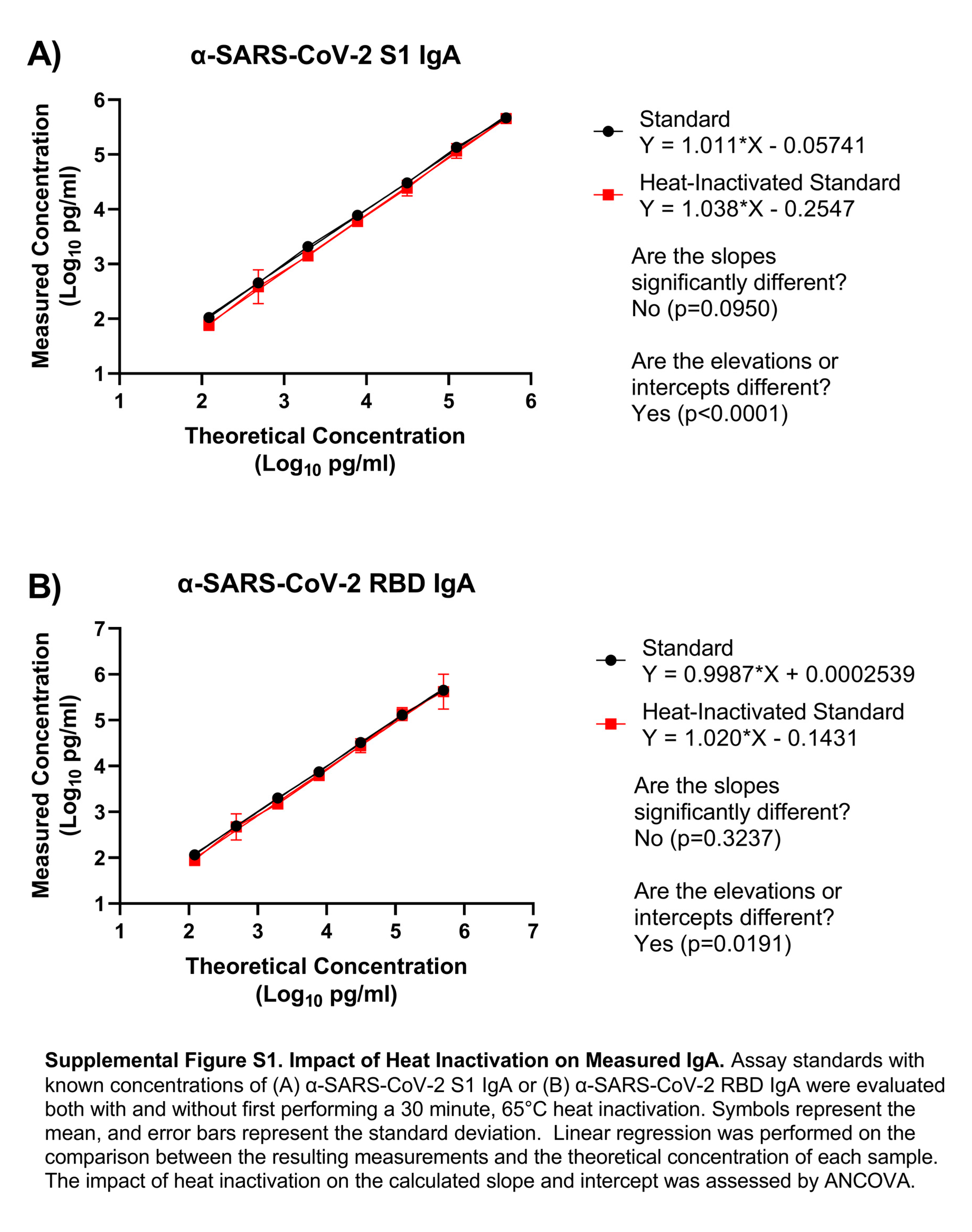

Supplement: Supplementary file 1 [file viruses-14-02071-s001.zip › Supplementary Figure S1 - Heat Inactivation.tif]

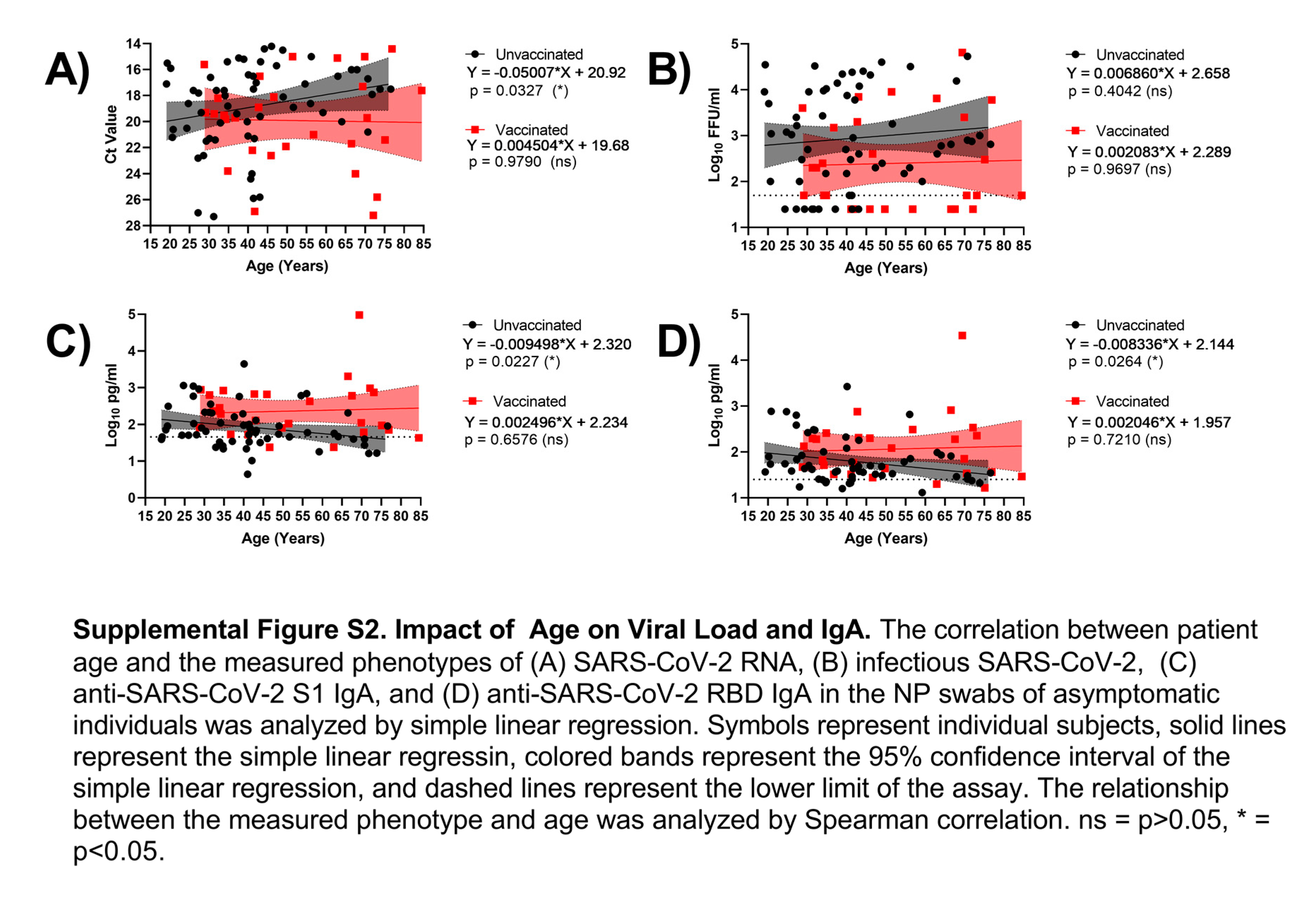

Supplement: Supplementary file 1 [file viruses-14-02071-s001.zip › Supplementary Figure S2 - Age.tif]
